# Characterization of *SQUAMOSA*-like genes in *Gerbera hybrida*, including one involved in reproductive transition

**DOI:** 10.1186/1471-2229-10-128

**Published:** 2010-06-25

**Authors:** Satu Ruokolainen, Yan Peng Ng, Suvi K Broholm, Victor A Albert, Paula Elomaa, Teemu H Teeri

**Affiliations:** 1 Gerbera Laboratory, Department of Agricultural Sciences, P.O. Box 27 (Latokartanonkaari 7), FIN - 00014 University of Helsinki, Finland; 2 Biomedicum Helsinki, P.O. Box 63 (Haartmaninkatu 8), FIN-00014 University of Helsinki, Finland; 3 Department of Biological Sciences, University at Buffalo (SUNY), Buffalo, NY, 14260, USA

## Abstract

**Background:**

The flowering process in plants proceeds through the induction of an inflorescence meristem triggered by several pathways. Many of the genes associated with both the flowering process and floral architecture encode transcription factors of the MADS domain family. Gerbera, a member of the sunflower family, Asteraceae, bears compressed inflorescence heads (capitula) with three different flower types characterized by differences in both sexuality and floral symmetry. To understand how such a complex inflorescence structure is achieved at the molecular level, we have characterized the array of Gerbera MADS box genes. The high number of *SQUAMOSA*-like genes in Gerbera compared to other model species raised the question as to whether they may relate to Gerbera's complex inflorescence structure and whether or not a homeotic A function is present.

**Results:**

In this paper we describe six Gerbera genes related to the *SQUAMOSA/APETALA1/FRUITFULL *genes of snapdragon and Arabidopsis. Based on phylogenetic analysis of the entire gene lineage, our data indicates that *GSQUA1 *and *GSQUA3 *are members of the *SQUA*/*AP1 *clade, while *GSQUA2*, *GSQUA4*, *GSQUA5 *and *GSQUA6 *are co-orthologs of the Arabidopsis *FUL *gene. *GSQUA1/GSQUA3 *and *GSQUA4/GSQUA5/GSQUA6*, respectively, represent several gene duplication events unknown in the model systems that may be specific to either Gerbera or Asteraceae. *GSQUA *genes showed specific expression profiles. *GSQUA1*, *GSQUA2*, and *GSQUA5 *were inflorescence abundant, while *GSQUA3*, *GSQUA4*, and *GSQUA6 *expression was also detected in vegetative organs. Overexpression of *GSQUA2 *in Gerbera led to accelerated flowering, dwarfism and vegetative abnormalities, all new and specific phenomena observed in transgenic Gerbera plants with modified MADS box gene expression.

**Conclusions:**

Based on expression patterns, none of the Gerbera *SQUA*-like genes are likely to control flower organ identity in the sense of the floral A function. However, our data shows that the *FUL*-like gene *GSQUA2 *plays a vital role in meristem transition. The roles of other *GSQUA*-genes in Gerbera floral development are intriguing, but require still further study.

## Background

*Arabidopsis thaliana *has been the principal model plant for molecular developmental studies of flowers for two decades. Several traits of Arabidopsis contribute to its attractiveness as a model system. However, not all phenomena in angiosperm flower development are present in Arabidopsis, and some processes are in fact specific to Arabidopsis or its close relatives (reviewed in [1]). Thus, extrapolating floral developmental paradigms from Arabidopsis to other flowering plants is not always straightforward [1-3]. To obtain a broader understanding of floral development, studies on species representing a broad taxonomic distribution are necessary. Our research interest has focused on floral development in *Gerbera hybrida*, a model species of the sunflower family (Asteraceae). Gerbera inflorescences consist of hundreds of flowers, which can be divided into three different types based on their size, sex, and position in the inflorescence. We have previously shown that many basic principles of floral development apply to Gerbera [4], but that in addition, Gerbera has special features of its own [5,6]. For example, the B and C functions of the ABC model of flower development [7] are applicable to Gerbera, but the A function has remained elusive.

Based on the ABC model, A function genes are involved in determining sepal and petal identity by repressing C function in whorls one and two [7]. *Arabidopsis *has two A class genes *APETALA1 *and *APETALA2 *(*AP1*, *AP2*) [8-12]. *AP1 *is a MADS box gene, as are the majority of the ABC function genes [12], while *AP2 *is a member of the *AP2/ERF *ethylene response family. Both *AP1 *and *AP2 *act as A function genes, but they also have several other functions (reviewed in [1]). *AP1 *has been shown to fulfil a dual function in specifying *Arabidopsis *sepal and petal identity as well as affecting floral meristem development [9,13]. *AP1 *acts closely together and partially redundantly with other inflorescence architecture genes, *CAULIFLOWER *(*CAL*) and *FRUITFULL *(*FUL*) [14]. Despite attempts to establish similar functions for related genes in other plant species, success has been limited. For example, the *Antirrhinum SQUAMOSA *(*SQUA*) gene plays a role in inflorescence meristem development but does not affect floral organ identity [15]. A similar function has been shown for the related gene *Antirrhinum DEFH28*, which is not involved in determination of sepal and petal identity [16]. Several plant species appear to have genes closely related to *AP1*, but apparently none have similar functions in specifying sepal and petal identity [17-22]. The pea (*Pisum sativum*) gene *PEAM4 *seems to be the closest to *AP1 *in function and has been suggested to be a functional homologue of *AP1 *[23] based on similar expression pattern and floral phenotype. However, several authors [1,24-26] have been inclined to suggest that the entire concept of an A function might be specific to Arabidopsis and perhaps other Brassicaceae.

In addition to previously characterized Gerbera MADS box genes [4-6], we have recently identified several Gerbera genes similar to *AP1*, *FUL *[9,11] and *SQUA *[15]. *AP1 *and *SQUA *are often described as A function genes, but only *AP1 *has characteristics of a homeotic selector gene. *AP1 *and *SQUA *do, however, play strong roles in defining floral meristem identity, together with the genes *LEAFY *in Arabidopsis and *FLORICAULA *in snapdragon [27,28].

Here, we analyze the expression and phylogenetic position of six Gerbera genes, *Gerbera SQUAMOSA-LIKE1-6 *(*GSQUA1-6*), which are closely related to *AP1*, *SQUA*, and *FUL*. Our data indicate that none of the *GSQUA *genes are, by themselves, likely to play a role in defining floral organ identity in the sense of the A function of the floral ABC model [7]. However, *GSQUA2 *does function as a strong positive regulator of meristem transition in Gerbera. Overexpression of *GSQUA2 *in transgenic Gerbera results in an early flowering dwarf phenotype, which displays abnormal vegetative architecture.

## Results

### Isolation and phylogenetic analysis of the *Gerbera hybrida GSQUA *genes

*GSQUA1 *was isolated earlier by low stringency screening of an inflorescence cDNA library using a spruce MADS box gene probe, and was so named based of its sequence similarity to *SQUA *of *Antirrhinum *[4,15]. PCR amplification using a degenerate MADS-box specific primer yielded three additional partial sequences of Gerbera *SQUA*-like genes: *GQUA2*, *GSQUA3*, and *GSQUA4*. Two more *SQUA*-like genes, *GSQUA5 *and *GSQUA6*, were identified from a Gerbera EST collection [29]. Full length cDNA sequences were recovered using 5' and 3' RACE for all *GSQUA *genes except for *GSQUA4*.

**Figure 1:**
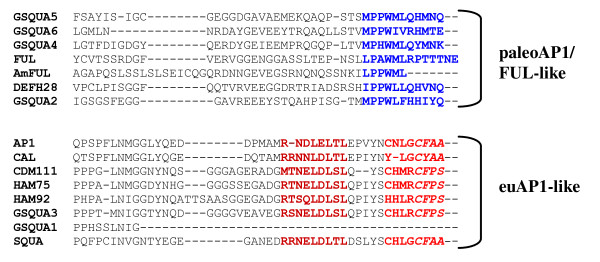
**Alignment of C-terminal ends of AP1-like proteins**. Gerbera GSQUA proteins were aligned with closely related proteins from Arabidopsis (AP1, P35631; FUL, Q38876; CAL, Q39081), snapdragon (SQUA, Q38742; DEFH28, Q941M9, AmFUL, Q7XBN7), chrysanthemum (CDM111, Q84LD6), and sunflower (HAM75, Q8RVR0; HAM92, Q84LC0). The paleoAP1/FUL protein motif is shown in blue [2,30]. EuAP1-like proteins contain both an acidic domain (shown in dark red), which has been shown to have transcriptional activity in yeast [86], and a farnesylation motif (shown in red italics) at their C termini [35]. The whole domain marked red represents the euAP1 motif according to [30].

In Arabidopsis, the A function/meristem-identity gene *AP1 *and the fruit function/meristem-identity gene *FUL *share a high degree of sequence similarity despite their partially different functions [9,11,14]. The C termini of plant MADS domain proteins are variable, but within closely related groups, conserved protein motifs can be recognized. Both AP1- and FUL-like proteins are characterized by such motifs, the euAP1-motif for the former, and the paleoAP1- or FUL-motif for the latter [2,30]. Alignment of the predicted amino acid sequence of GSQUA2 with similar sequences from other plant species showed that GSQUA2 contains a protein motif similar but not identical to the paleoAP1/FUL-motif. The same motif was also recognizable in GSQUA4, GSQUA5 and GSQUA6. In contrast, GSQUA3 possessed a euAP1-motif (CFPS) that is divergent from the consensus motif (CaaX) [2,30], while still containing several conserved amino acids (Figure 1). In the previously isolated GSQUA1 protein [4] a euAP1-motif was not evident, but phylogenetic analysis (Additional files 1 and 2) nevertheless suggested a close relationship between GSQUA1 and GSQUA3. The deduced Gerbera GSQUA amino acid sequence alignments and the corresponding protein motifs are shown in Figure 1.

Phylogenetic analysis suggested that *GSQUA1 *and *GSQUA3 *are close paralogs, together co-orthologous to *AP1 *(and *SQUA*). Similarly, *GSQUA4*, *GSQUA5 *and *GSQUA6 *are co-orthologs of *FUL*, and *GSQUA2 *is phylogenetically close to the snapdragon gene *DEFH28*. Although interrationships among the *AP1/SQUA*, *DEFH28*, and *FUL *clades are not well supported in the phylogenetic analysis, the conserved C terminal motifs suggest that *GSQUA2/DEFH28/AmFUL *are *FUL*-like. The full maximum likelihood tree, based on our sequences added to the alignment of [2], is shown in Additional file 2. An alignment of *GSQUA *DNA sequences is shown in Additional file 1.

### RNA gel blots and *in situ *hybridization of *GSQUA *genes

**Figure 2:**
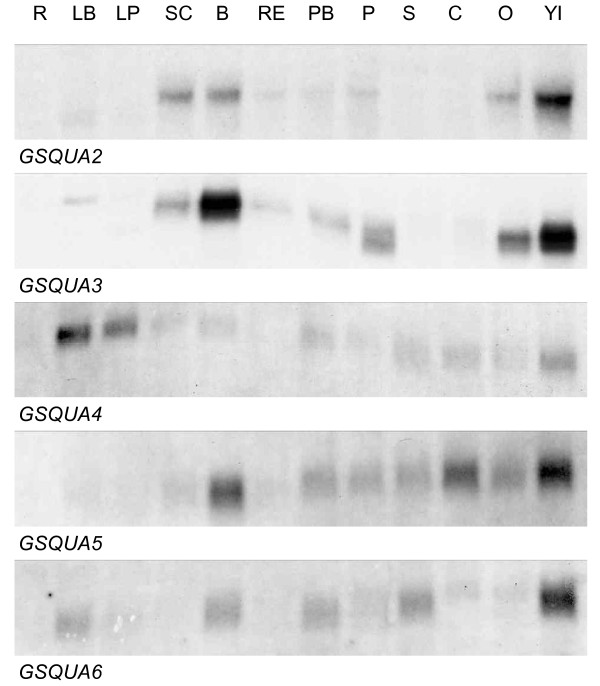
**Expression of *GSQUA *genes in various Gerbera tissues**. R, roots; LB, leaf blade; LP, leaf petiole; SC, scape; B, bracts; RE, receptacle; PB, pappus bristles; P, petals; S, stamen; C, carpel; O, ovary; YI, young inflorescence (6-16 mm in diameter).

Figure 2 summarizes the expression patterns of *GSQUA2-6 *at RNA gel blot level. Based on previous studies, *GSQUA1 *expression was in the young inflorescence, scape and bracts [4]. In addition to *GSQUA1*, the expression of *GSQUA2*, and *GSQUA5 *was restricted to floral tissues and no expression was detected in vegetative organs. Interestingly, *GSQUA3*, *GSQUA4 *and *GSQUA6 *also showed expression in leaves, in addition to expression in floral and inflorescence-derived organs. None of the studied *GSQUA *genes were expressed in Gerbera roots. At the level of single (ray) flowers at relatively late developmental stages, *GSQUA2 *and *GSQUA3 *transcripts were most abundant in whorls one and two, while *GSQUA4*, *GSQUA5*, and *GSQUA6 *were expressed in all floral whorls (Figure 2). Different developmental stages of Gerbera ray flower petals (see [31]) were screened by RNA gel blot hybridization to ascertain whether expression levels of *GSQUA *genes varied over time. The expression levels of *GSQUA3 *and *GSQUA5 *did not vary during ray flower petal development, whereas the expression of *GSQUA4 *was barely detected during ray flower petal development, and both *GSQUA2 *and *GSQUA6 *showed differential expression. *GSQUA2 *expression was stronger during early stages (1,2,3) and faded noticeably toward later developmental stages (4,5,6,7,8,9,10,11). *GSQUA6 *expression displayed a pattern opposite to that of *GSQUA2*; its expression grew stronger toward later developmental stages (8,9,10,11) (Additional file 3).

**Figure 3:**
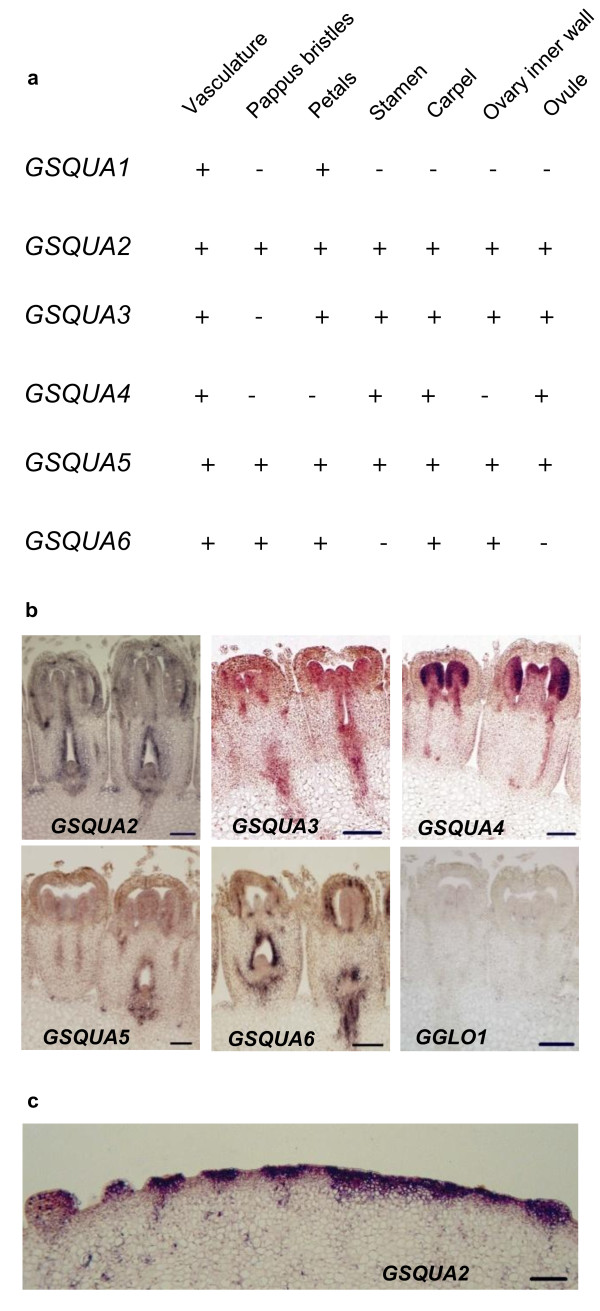
**RNA *in situ *expression analysis of *GSQUA *genes**. (a, b) Developing Gerbera inflorescence (diameter 9-17 mm). Results for *GSQUA1 *were previously published in [4]. Generally, many *GSQUA *genes are widely expressed during inflorescence development. Expression in vasculature of the receptacle in addition to floral organs is a common feature. Both *GSQUA2 *and *GSQUA5 *are expressed in all floral organs while especially *GSQUA4 *has more specific expression pattern. (c) The expression of *GSQUA2 *starts early and marks the location of emerging individual flowers in the developing young inflorescence (diameter 6 mm).

To localize *GSQUA *expression during the early stages of inflorescence development, a more detailed RNA *in situ *hybridization analysis of young, developing Gerbera inflorescences (diameter 6-17 mm) was performed (Figure 3). In general, *GSQUA *genes studied here showed a wide range of expression patterns. In fact, the vasculature of the capitulum receptacle was the only common location where all of the *GSQUA *genes were expressed. In contrast to other *GSQUA *genes, *GSQUA1 *was entirely restricted to the vasculature of the capitulum receptacle and petals [4].While *GSQUA2 *and *GSQUA5 *were found to be expressed in all parts of the inflorescence, *GSQUA3 *and *GSQUA6 *displayed a slightly narrower expression pattern at the inflorescence level. *GSQUA4 *was expressed only in the reproductive organs in addition to the vasculature (Figure 3a, b, ). Figure 3b shows examples of developing individual ray flowers, while the summary in Figure 3a is based on larger number of *in situ *hybridizations. *GSQUA2 *expression was also seen in the receptacle between the emerging individual flowers (inflorescence size 6 mm, visible also in inflorescence size 14 mm) and petal expression was localized to the adaxial surfaces (Figure 3b). The location of emerging flowers in the developing inflorescence was marked by strong *GSQUA2 *expression even before clear anatomical differentiation was visible at the center of the capitulum (inflorescence diameter 6 mm) (Figure 3c).

### Phenotypic changes in *GSQUA2 *overexpression lines

**Figure 4:**
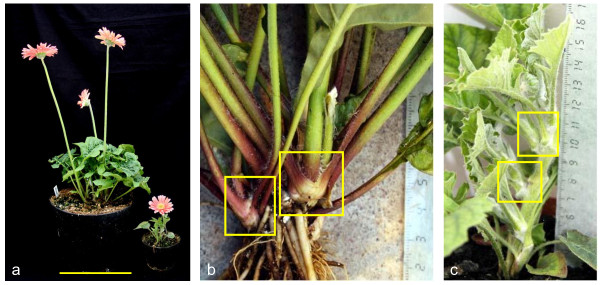
**Transgenic Gerbera overexpressing *GSQUA2***. (a) Gerbera overexpressing *GSQUA2 *displays dwarf phenotype and flowers early. Wild type cultivar 'Terra Regina' on the left side. Scale bar, 20 cm. (b) Normal Gerbera growth habit is sympodial, the leaves forming a rosette-like structure consisting of tightly packed sympodial units. (c) In plants overexpressing *GSQUA2*, vegetative axes between sympodial units are strongly elongated compared to the wild type plant. Examples of sympodial units are framed in yellow squares.

For functional analysis, we were only able to obtain clear and consistent phenotypes by overexpressing *GSQUA2*. Transformation of Gerbera with *GSQUA2 *under the 35S promoter yielded five lines strongly overexpressing *GSQUA2 *and one line with weaker overexpression, which correlated with milder phenotypic changes (Additional file 4). Compared to the non-transformed Gerbera cultivar 'Terra Regina', all strong overexpression lines showed altered vegetative growth very early in development. The posture of the plants was upright, with leaves curving adaxially. The normal growth habit that leads to a tight rosette-like arrangement of leaves in Gerbera [32] was loosened, with the segments/vegetative axis of the stem strongly elongated. Inflorescences started to form after only two months in the greenhouse whereas the wild type cultivar 'Terra Regina' typically reaches the flowering stage after 6 months (Figure 4). Root formation of the overexpression plants was poor. The plants were susceptible to molds in greenhouse conditions and they typically died after forming only a few inflorescences. Transformants grown in more controlled and contamination-free growth chamber conditions survived for longer periods of time. The general appearance of overexpression lines of *GSQUA2 *was unstable due to their aberrant architecture, and they required support to remain upright. One milder phenotype was also observed (TR3). This line was not as dramatically dwarfed, but was clearly smaller and more delicate in structure, both vegetatively and inflorescence-wise, as compared to non-transgenic plants. RNA gel blot analysis showed strong expression for *GSQUA2 *in the inflorescence, but overexpression in leaves was weaker compared to overexpression lines showing the dwarfed phenotype (Additional file 4).

The number of flowers in the inflorescence of *GSQUA2 *overexpression lines was reduced compared to wild type. Non-transformed Gerbera 'Terra Regina' inflorescences, grown side by side with the transformants in the greenhouse, contained on average about 900 individual flowers. The *GSQUA2 *overexpression lines produced on average only 420 flowers in their inflorescences (Table 1).

**Table 1:** The number of individual flowers in wild type Gerbera 'Terra Regina' inflorescence vs. *GSQUA2 *overexpression lines

Inflorescence	Number of flowers/inflorescence	Average
wt 'Terra Regina'	882, 830, 965, 1001, 859	907,4
CaMV 35S :: *GSQUA2*	534, 457, 535, 371, 202	419,8*

* Significant at P < 0.001 (t-test)

Inflorescences upregulated for *GSQUA2 *contain less than 50% of flowers of wild type *Gerbera*.

Inspection of *GSQUA2 *overexpression lines with stereomicroscopy or scanning electron microscopy showed no homeotic changes in floral organs of any flower type (data not shown). However, petals of all flower types were shorter compared to the wild type petals, which is congruent with dwarfism and the overall smaller size of the inflorescence. Additionally, inflorescence color differed from wild type in being paler. Despite three transgenic lines producing antisense RNA for *GSQUA2*, no silencing of the endogenous *GSQUA2 *was observed.

## Discussion

### The *GSQUA *subfamily of MADS box genes contains at least six members in Gerbera

In addition to the previously published Gerbera *SQUA*-like genes, *GSQUA1 *[4], *GSQUA5 *and *GSQUA6 *[29], we isolated three new sequences, *GSQUA2, GSQUA3 *and *GSQUA4*. The number of *GSQUA *genes is large compared to most other plant species and it is tempting to relate this diversity to the complex structure of the Gerbera inflorescence [33]. Arabidopsis *AP1 *and *FUL*, which function in sepal and petal, fruit, and meristem development [9,11,14], are closely related to *GSQUA*s at the sequence level. The relationship of the two Arabidopsis proteins has been further analyzed by [2] and [30], and they described conserved C-terminal protein motifs (euAP and paleoAP/FUL) in a number of AP1- and FUL like sequences. Identification of these motifs facilitates the classification of related proteins, since phylogenetic analysis of AP1- and FUL-like sequences is not always unambiguous. The paleoAP1/FUL-like protein sequences have a hydrophobic motif (L/MPPWML), which is not found in euAP1-like sequences. EuAP1-like sequences in turn have two conserved motifs, a transcription activation domain RRNaLaLT/NLa (where 'a' stands for an acidic amino acid [2]) and a farnesylation signal CaaX (where C is Cys, 'a' is an aliphatic amino acid, and X is Cys, Met, Ser, Ala, or Glu [34]) that terminates the protein. A farnesylation motif generally directs proteins to a membrane [34], but the role of farnesylation in plant proteins might be more diverse [35-37]. In the case of transcription factors, this function could be part of post-transcriptional regulation, or necessary for protein complex formation [36]. AP1 has been shown to be farnesylated *in planta*, but membrane localization was not observed [36]. Not all euAP1-like proteins possess this farnesylation signal, however, and thus it may not be an essential part of the protein function [1,23].

Based on the presence of conserved C terminal protein motifs, GSQUA3 can be classified as belonging to the euAP1-like proteins, while GSQUA2, GSQUA4, GSQUA5 and GSQUA6 harbor a paleoAP1/FUL-like protein motif at the C terminus of their amino acid sequence (Figure 1). GSQUA1 does not possess a recognizable protein motif of either type at its C terminal end, but phylogenetic analysis places it close to GSQUA3 (Figure 2). In fact, the GSQUA1 sequence terminates 16 amino acids before the expected euAP1 protein motif. Furthermore, the GSQUA3 protein sequence contains the transcriptional activation domain RSNELDLSL, but no strong transcriptional activation was seen in yeast assays [38]. The motif differs slightly from the consensus motif RRNaLaLT/NLa [2], the second arginine being replaced by serine in GSQUA3 and threonine or asparagine being substituted for serine. The functional relevance of these changes is not clear. Despite the close sequence similarity in the C terminal domain of GSQUA3 to related proteins such as AP1 and SQUA [9,11,15], the farnesylation domain of GSQUA3 (CFPS) differs from the most common version of the motif, CFAA/T [35], which is found in many plant SQUA-like proteins [2]. EuAP1-protein motifs similar to Gerbera GSQUA3 are also present in related protein sequences of other species in Asteraceae, including sunflower (*Helianthus annuus*) and Chrysanthemum (*Dendrathema grandiflorum*) (HAM75, HAM92, CDM111) [21,39]. Still, these Asteraceae specific variants are within the definition of the farnesylation motif CaaX [35]. The current definition of the consensus motif is possibly too narrow, and as more plant species are studied in detail, the farnesylation consensus motif may require redefinition.

A detailed phylogenetic analysis of *GSQUA2, GSQUA3, GSQUA4, GSQUA5 *and *GSQUA6 *produced results in line with the relationships suggested by analysis of C terminal protein motifs. The maximum likelihood tree suggests that *GSQUA2 *may be orthologous to the snapdragon gene *DEFH28*, which is involved in the regulation of floral meristem identity and fruit development [16]. Both of these *DEFH28 *functions are similar to *FUL *of Arabidopsis, and the authors concluded that *DEFH28 *most likely represents the ortholog of *FUL*. However, this interpretation was later challenged by [2] based on the discovery of *AmFUL*, which, according to phylogenetic and protein motif analysis more likely represents the snapdragon gene orthologous to *FUL*. Unfortunately, *AmFUL *has not been further characterized. *GSQUA2 *does share the early flowering function of *DEFH28*, however. A potential role of *GSQUA2 *in fruit development was not studied in this work.

Previous and recent studies on *FUL*-like genes further distinguish two groups [1,2,40]. *FUL *and *AmFUL *belong to the *euFUL *group [41,2], while *AGL79 *and *DEFH28 *belong to *euFULII *group [16,40]. Based on the phylogenetic analysis *GSQUA4*, *GSQUA5*, and *GSQUA6 *genes are closer to the *euFUL *group, while *GSQUA2 *belongs to the *euFULII *group.

*GSQUA1 *[4] and *GSQUA3 *appear to be recent paralogs and are co-orthologous to *SQUA *of snapdragon [15]. Similarly, *GSQUA4*, *GSQUA5 *and *GSQUA6 *are coorthologous to *FUL *of Arabidopsis [14,41].

### The expression patterns for *GSQUA *genes do not support a homeotic A function

All *GSQUA *genes, despite being closely related, exhibit different expression patterns at the vegetative and floral organ levels. However, none of the *GSQUA *genes investigated share the expression pattern of Arabidopsis *AP1 *or snapdragon *SQUA *in the sense that they would be particularly abundant in floral whorls 1 and 2 (sepals and petals) in early stages of development. In general, at earlier developmental stages, expression domains of *GSQUA*s are widespread at the inflorescence level, with the exception of *GSQUA4*, which is expressed in reproductive organs and in the vasculature of the capitulum receptacle (Figure 3a, b). Only later in floral development *GSQUA2 *and *GSQUA3 *are weakly expressed in sepals and petals (Figure 2). The expression in vasculature is common among all *GSQUA *genes studied here. Expression in vasculature is also known for *FUL *[41] and *AmFUL *[2], but vascular expression is not a uniform trait for *euAP1*-, *euFUL*- or *euFULII*-like genes. This expression pattern may reflect a function in developing vascular bundles, but the phenomenon has not been extensively discussed previously and its functional significance for *GSQUA *genes remains unclear.

The broad expression pattern of *GSQUA2 *during early stages of ray flower development resembles what has been previously reported for *FUL *and other *FUL*-like genes, and contrasts with the expression of *AP1*, which is confined to the first two whorls [10]. *FUL*-like genes are commonly expressed in the carpel [21,42-45], meristems [13,41] and vegetative tissues, including bracts [18,21,43,46]. Expression has also been observed in the inflorescence [18,19,21,47,48], floral meristems [19,49], stamens [17,45], and perianth organs [17,42,43,45]. For some species, expression has been visible in all floral whorls [45,50]. The expression pattern for Arabidopsis *FUL *is biphasic, which is in accordance with its early (floral meristem identity) and late (silique development) functions in reproductive development [14,41].

The functional role of *FUL *in fruit development was first detected in Arabidopsis mutant lines lacking *FUL *expression. Gerbera does not bear a fruit similar to Arabidopsis; its ovary position is inferior as opposed to superior in Arabidopsis and the fruits (achenes) are indehiscent. Thus the late function for GSQUA2 might be entirely different (like *DEFH28 *in snapdragon; [16]) or lacking completely. The most dramatic phenotypic effects in 35S::*FUL *lines are cell type changes in valve margins and the outer replum, which lead to developmental failure of the dehiscence zone and eventually to indehiscent fruit [14]. Interestingly, *GSQUA2 *expresses strongly in ovary inner walls and the ovule (Figure 3), so despite the fact that no homeotic changes in *GSQUA2 *overexpression lines were visible in ovaries and ovules at the relatively late developmental stage 8, a role for *GSQUA2 *in Gerbera fruit development, possibly at the level of cell differentiation, cannot be ruled out.

### *GSQUA2 *is involved in meristem transition

Among the several related *GSQUA *genes of Gerbera, only *GSQUA2 *lent itself to further functional characterization based on transgenic Gerbera lines overexpressing the gene. Several transgenic lines both for *GSQUA3 *and *GSQUA5 *were generated and analyzed for overexpression and downregulation, but no consistent floral phenotypes were observed. Both genes, *GSQUA2 *and *FUL*, seem to share the same function of meristem identity determination in early floral development, but the inflorescence abundance of *GSQUA2 *expression distinguishes it from *FUL*, as *FUL *is expressed also in vegetative parts of Arabidopsis [13]. However, when *GSQUA2 *is ectopically expressed throughout Gerbera tissues, dramatic vegetative changes such as dwarfism and vegetative axis elongation appear. Gerbera growth habit is sympodial with very short, leafy lateral shoots forming the sympodia. Typically, the sympodial rhizome forms 7-24 leaves before the first inflorescence is formed by the apical meristem. Two inflorescences are formed per one vegetative shoot, the second inflorescence being formed in the axil of the uppermost leaf primordium. The vegetative axis continues to develop in the axil of the second leaf primordium. The fully-formed axis grows 2-8 leaves before forming a terminal inflorescence, a lateral inflorescence, and again a vegetative shoot, the growth cycle being iterative [32]. The vegetative axis between lateral shoots is very short and the lateral shoots form a tightly packed entity. However, in plants overexpressing *GSQUA2*, the vegetative axis between lateral shoots is strongly elongated compared to wild type Gerberas (Figure 4). The poor root formation of the overexpression lines may be to ectopic expression of *GSQUA2 *under the 35S promoter, which interferes with the normal root development and is thus not necessarily informative of the gene's normal function.

Overexpression lines of *GSQUA2 *flower substantially earlier than wild type plants, which suggests this gene to be involved in floral meristem transition. The strong localized expression of *GSQUA2 *in emerging flower primordia at the early stages of flower development also supports this hypothesis (Figure 3c). Despite of the strong expression in overexpression lines, only minor morphological changes, such as reduced petal size and color, were detected at the level of individual flowers. At the inflorescence level, however, a considerably reduced number of flowers was observed, since the overexpression lines for *GSQUA2 *contained only half the number of flowers in their inflorescences as non-transgenic Gerbera. A similar phenomenon was reported with birch *BpMADS4 *overexpression lines [51], and may relate to accelerated development, including accelerated consumption of the inflorescence meristem.

In wheat and ryegrass, the *AP1*-like MADS-box gene *VRN1 *is expressed in vegetative tissues and has been suggested to control the transition to flowering [52,53]. Based on the vegetative expression pattern, *GSQUA3*, *GSQUA4 *and *GSQUA6 *are Gerbera candidates for this kind of function, but at least for *GSQUA3 *we have data that its ectopic expression does not cause early flowering.

In Arabidopsis, accelerated flowering is regularly observed when different MADS-box genes are overexpressed, including those not directly related to flowering time [54-63]. In Gerbera, all overexpression lines with MADS box genes other than *GSQUA2 *have retained their normal vegetative size and flowering time, although many have displayed homeotic or meristem identity changes in the inflorescences [4-6].

### GSQUA proteins interact with other Gerbera MADS domain proteins

AP1/SQUA-like MADS domain proteins have been suggested to function as mediators of higher order complex formation, acting as 'bridge proteins' and facilitating the formation of protein quartets [64,65]. However, based on pairwise assays [38], GSQUA proteins seem unlikely to function as interaction mediators in Gerbera, since their interaction capacity appears to be limited [38]. This feature distinguishes all GSQUA proteins from the closely related Petunia protein FBP29. FBP29 is capable of interacting with several MADS domain proteins of different functional classes [43]. Moreover, other FUL-like proteins from Petunia, PFG and FBP26, show more extensive interaction capacity than the studied GSQUA proteins [42,43]. Also Arabidopsis FUL was shown to be active in multiple protein-protein interactions [66]. GSQUA2 was found to interact with three other Gerbera MADS domain proteins in a screen of fourteen proteins, whereas GSQUA1 and GSQUA3 proteins interacted with only two other proteins, all partners being members of the SEP-like GRCD family of Gerbera proteins. GSQUA5 remained inactive in pairwise assay showing no interaction with any tested Gerbera proteins. The most interesting GSQUA2 specific partner is GRCD2, a pleiotropically active Gerbera SEP-like protein with functions in carpel identity, meristem identity and inflorescence determinacy [6]. Interestingly, when GSQUA2 and GRCD2 were combined in yeast, a strong autoactivation function emerged - separately, neither of the proteins show transcriptional activation. This function of the GSQUA2/GRCD2 dimer could reflect its importance in Gerbera floral development. Both GSQUA2 and GRCD2 are co-expressed in young inflorescences and their expression patterns are overlapping [6], rendering the interaction feasible also *in planta*.

When assaying for higher order complex formation, GSQUA proteins showed greater activity. Together with the Gerbera B function dimer GGLO1/GDEF2, and when combined with a Gerbera SEP-like GRCD protein and with a C function GAGA protein, all GSQUA proteins showed activity [38]. While GSQUA proteins did not interact with each other in the pairwise assays, addition of a GRCD protein made some complexes with two GSQUA proteins stable in yeast.

Even as interaction of GSQUAs with E function proteins (GRCD4 and GRCD5, pairwise) or with B function proteins (GGLO1/GDEF2, threesome) can be seen as consistent with a homeotic A function for GSQUAs, interaction with C function proteins (GAGA1 and GAGA2, threesome with GRCDs) is not. In Arabidopsis, expression of *AP1 *(with homeotic A function) is excluded in cells where the C-function gene *AGAMOUS *(*AG*) is expressed [67]. AP1 alone does not repress the C function in whorls one and two, but rather acts together with the non-MADS proteins LEUNIG and SEUSS [68,69] in a complex including other MADS domain proteins, AGL24 and SVP [68]. However the *AG *gene has functions beyond the floral homeotic one in Arabidopsis. *AG *is known to control the meristematic state of flower primordia and to downregulate the meristem organizing gene *WUSCHEL *together with unknown factors [70,71] which in Petunia are MADS domain proteins [72].

It is tempting to relate the large number of *SQUA*-like genes in Gerbera to the complex structure of the inflorescence in Asteraceae. At least some interactions for homologous Chrysanthemum MADS domain proteins are similar to the Gerbera proteins. CDM41, which is closely related to GSQUA proteins, interacts with Chrysanthemum CDM44, which is homologous to SEP3 of Arabidopsis [21]. This interaction is similar to GSQUAs' interaction with GRCD4 and GRCD5. In yeast three-hybrid assay, CDM41 combined with the Chrysanthemum B protein heterodimer (CDM86 and CDM115), and the complex was active, as are Gerbera complexes with a GSQUA protein and the B protein dimer. Sunflower (*Helianthus annuus*) also contains several genes closely related to *AP1 *and *FUL *[39]. Obviously duplication of this lineage of genes has also taken place in sunflower. Perhaps gene duplication and divergence in the *SQUA/AP1/FUL *gene lineage has participated, together with the unique diversity in *TCP *family transcription factors [73] to help shape the complex Asteraceae inflorescence.

## Conclusions

Gerbera has an array of SQUA-like genes, which can be classified either as euAP1-like, or as FUL-like [2,30]. However, none of these genes appear to act as an A function gene in the sense of the classical ABC model [7]. Based on these results, Gerbera can be added to the growing list of plant species that lack the A function comparable to Arabidopsis. *GSQUA2 *is intimately involved in the regulation of meristem transition in Gerbera as overexpression of *GSQUA2 *led to accelerated flowering. The role of *GSQUA1*, *GSQUA3*, *GSQUA4*, *GSQUA5*, and *GSQUA6 *in the floral development of Gerbera requires further study. The complex inflorescence structure and the high number of Gerbera *GSQUA*-like genes lead to a temptation to associate these two phenomena, but verifying this hypothesis requires more research.

## Methods

### Identification of Gerbera *GSQUA *genes

*GSQUA2, GSQUA3 *and *GSQUA4 *were identified using reverse transcription PCR with inflorescence mRNA as a template. The 5' primer E0364 (GCG GAG CTC GAG TTA AGA GRA TAG ARA ACA , where R = A/G) was designed based on previously published alignment of the MADS domain from several plant species, including Gerbera [4,74]. The 5' end of the primer contained two restriction enzyme recognition sites (for *Sac*I and *Xho*I) to aid cloning. For the 3' end, an anchored oligo-d(T) primer (G ACC ACG CGT ATC GAT GTC GAC TTT TTT TTT TTT TTT TV, V = G/C/A) (Boehringer Mannheim 5'/3' RACE kit 1734792) was used. This primer contained three restriction enzyme cut sites (*Mlu*I, *Cla*I, *Sal*I) at its 5' end. The cDNA was synthesized from Gerbera inflorescence mRNA (pooled RNA sample, inflorescence sizes 10-13 mm in diameter) (Boehringer Mannheim kit 1483188). Taq DNA polymerase (Promega), 50 pmols of both primers and Gerbera inflorescence cDNA were used in a standard PCR reaction with 30 cycles. In an agarose gel, the result of the PCR showed several clear-cut bands of DNA. Four bands (estimated sizes 820 bp, 780 bp, 700 bp, and 550 bp) were isolated from the gel, ligated into the pBluescriptII SK + vector and sequenced. The largest fragment contained nearly full length sequences for *GSQUA2*, *GSQUA3 *and *GSQUA4*. *GSQUA5 *and *GSQUA6 *were identified in the Gerbera EST collection previously described [29]. *GSQUA5 *was recovered as a full-length cDNA from the EST collection, but *GSQUA6 *was about 100 nucleotides short at the 5' end of the gene.

### Isolation of full length sequences

Amplification with the E0364 primer left MADS box genes short of sequences encoding the amino acids in the N terminus of the protein. The missing sequences were amplified by the 5' RACE method [75] (5'/3' RACE kit, Boehringer Mannheim, cat. no. 1734792). Gene specific 5' RACE primers were designed from the intervening region between the MADS and the K boxes to ensure sufficient specificity. New cDNA was synthesized from Gerbera inflorescence mRNA (pooled RNA sample, inflorescence sizes 10-13 mm) (Boehringer Mannheim kit cat. no. 1483188). For each reaction, a band of approximate size of 500 bp was isolated from an agarose gel and ligated into the pGEM-T Easy vector (Promega). The missing 3' sequences of *GSQUA3 *and *GSQUA4 *were amplified using the same RACE kit. Finally, each full-length cDNA sequence was reamplified using gene specific 5' and 3' primers, ligated into the vector pBluescriptII SK + and verified by sequencing. Full-length sequences were obtained by 5' and 3' RACE methods for all *GSQUA *genes, except for *GSQUA4*, which lacks nucleotides encoding presumably about eight N terminal amino acids.

### Phylogeny reconstruction

For phylogenetic positioning of the *GSQUA *nucleotide sequences, we added them to the large data set used in [2]. The original data was kindly transmitted by A. Litt, and sequence abbreviations used by [2] apply to the present tree as well. The new alignment including Gerbera *SQUA*-like genes was made by hand, using the inferred amino acid sequences as a guide. The original *GSQUA1 *sequence in the [2] data matrix was deleted to avoid double representation. Phylogenetic analysis on the nucleotide data was performed using the maximum likelihood method, via the PHYML program [76], web interface [77]. 100 bootstrap resampling replicates were done to estimate support for the clades [78]. The options used with the PHYML web interface were the HKY molecular evolutionary model [79], transition/transversion ratio preset to 4, estimated proportion of invariant sites = 0.065, empirical nucleotide frequencies [f(A) = 0.32198, f(C) = 0.21318, f(G) = 0.24109, f(T) = 0.22375], 4 substitution rate categories, estimated gamma distribution parameter = 1.095, starting tree constructed using BIONJ [80], tree topology optimization using NNI and SPR tree rearrangement algorithms to search tree space, and branch length and rate parameter optimization.

### RNA gel blots

RNAs from different plant organs and from different stages of petal development (stages 1-11, see [31]) were isolated using Trizol (Invitrogen, cat. no. 11596-018) and quantified by spectrophotometer. Equal amounts (10 μg) of RNA were run in a 0.8% agarose gel as described by [31]. The rRNA bands were visualized by EtBr staining to record even loading of the gel. The RNA was blotted on Hybond-N membrane (Amersham Biosciences) and hybridized in the UltraHyb hybridization buffer (Ambion). For *GSQUA2*, a gene specific probe (260 or 320 bp) from the 3' UTR was used. The probe was labeled with [ ^32^P] dCTP and hybridized at + 42°C for 16 h. The membranes were washed with 1 x SSC, 0.1% SDS at + 42°C for 20 minutes. Subsequent washes were performed at + 65°C in the same buffer for 15 minutes, 1-2 times depending on the desired level of final activity. Films were exposed at -80°C. For *GSQUA3*, *GSQUA5*, and *GSQUA6*, full length probes (889 bp, 948 bp, and 812 bp) were used in hybridization due to unspecific hybridization patterns produced with shorter 3' probes. For *GSQUA4*, a longer probe of 450 bp was used due to problems with specificity. For RNA blots hybridized with longer probes, more stringent washing conditions with 0.2 x SSC, 0.1% SDS at + 65°C were applied, leading to increased specificity judged by simpler band patterns.

### *In situ *hybridization

*In situ *hybridization analysis was performed as described in [81] and [82]. *GSQUA2, GSQUA3, GSQUA4, GSQUA5 *and *GSQUA6 *gene specific *antisense *probes (250 bp, 385 bp, 300 bp, 187 bp and 235 bp from the 3' UTR) were prepared and quantitated using the DIG RNA labeling kit (Boehringer Mannheim cat. no. 11175025910) according to the manufacturer's instructions. Paraffin sections (10 μm thick) were mounted in 50% glycerol after hybridization. A 217 bp fragment of Gerbera *GGLO1 *from the 3' UTR [4] was used as a *sense *control in *in situ *hybridization.

### Plant material and transformation

*Gerbera hybrida *var. 'Terra Regina' was obtained from the commercial producer Terra Nigra, De Kwakel, the Netherlands. In the greenhouse, day length followed the natural day length during the summer season and was set to ten hours during the winter - day length is, however, not critical for Gerbera growth and flowering. The temperature was +16... + 18°C during nighttime and + 18... + 20°C during daytime. The plants were drip-irrigated and fertilized with NPK fertilizer (Kukka-Superex NPK 11-3-26, Kekkilä, Finland). The relative humidity was set for 65%. In growth chambers, temperatures were + 18°C at night and + 20°C during day, and the day length was set to 10 hours. For functional analysis, the full length *GSQUA *sequences were cloned under the CaMV 35S promoter in both *sense *and *antisense *orientation as described in [83]. Gerbera transformation was performed using an *Agrobacterium*-mediated gene transfer method as previously described [84,85].

## Authors' contributions

SR designed the experiments, carried out the experiments, analyzed the results and drafted the manuscript. YPN performed the RNA gel blot and *in situ *analyses for *GSQUA5 *and *GSQUA6*. SKB carried out the light microscopic and SEM analysis of the transformant lines for *GSQUA2*. VAA did the phylogenetic analysis, participated in the interpretation of the results and helped to draft the manuscript. PE participated in the design of the experiments, analysis of the results and helped to draft the manuscript. THT supervised the study, contributed to the design of the experiment, analysis of the results and helped to draft the manuscript. All authors read and approved the final manuscript.

## Supplementary Material

Additional file 1 ***GSQUA *nucleotide sequence alignment**. Nucleotide sequence alignment of the Gerbera *GSQUA *genes with other *APETALA1 *and *FRUITFULL *like genes. Click here for file

Additional file 2 **Phylogenetic tree of *SQUA*-like genes**. Phylogenetic analysis on the nucleotide data was performed using the maximum likelihood method. Click here for file

Additional file 3 **Expression of *GSQUAs *during ray flower petal development**. RNA gel blots showing the expression of *GSQUA2*, *GSQUA3*, *GSQUA4*, *GSQUA5*, and *GSQUA6 *at different stages of Gerbera ray flower development. Click here for file

Additional file 4 **Transgenic lines overexpressing *GSQUA2***. RNA gel blots showing *GSQUA2 *overexpression in transgenic lines. Click here for file
