# Supplementary figures and images for: Characterization of SQUAMOSA-like genes in Gerbera hybrida, including one involved in reproductive transition
Source: BMC Plant Biol. 2010 Jun 25;10:128. doi: 10.1186/1471-2229-10-128 (PMC3017819; doi:10.1186/1471-2229-10-128)

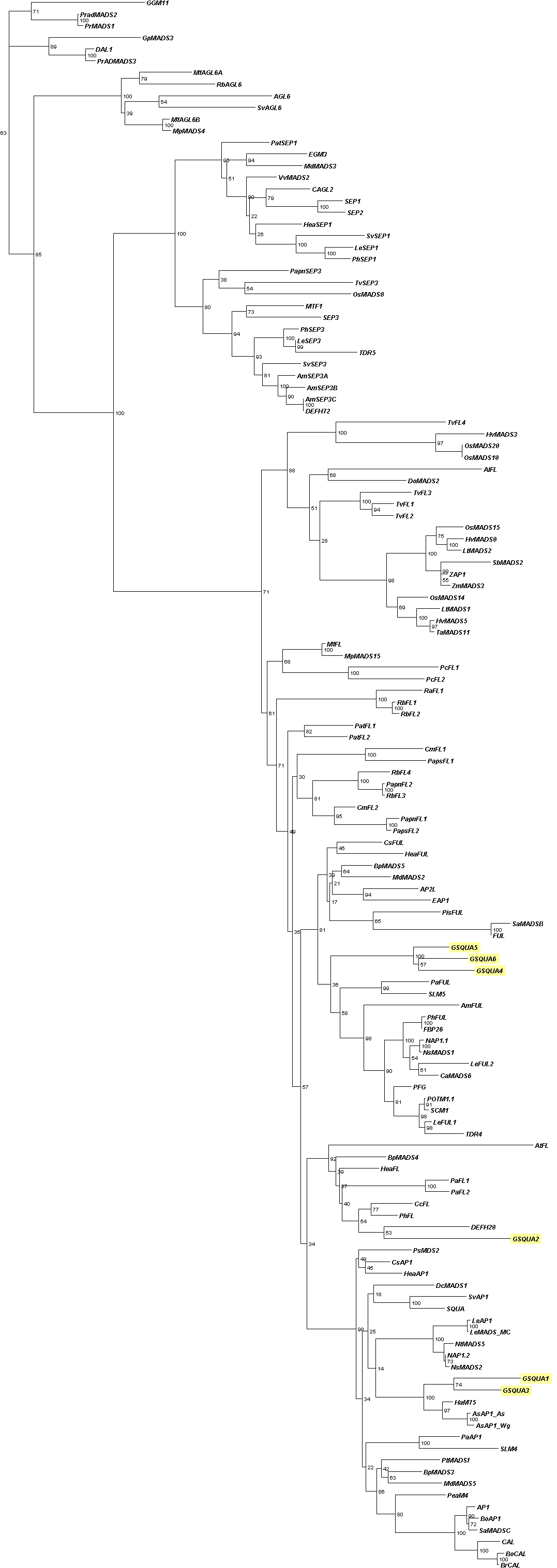


**Additional figure 2**. Phylogenetic tree of *SQUA*-like genes.

Supplement: Additional file 2 — Phylogenetic tree of SQUA-like genes. Phylogenetic analysis on the nucleotide data was performed using the maximum likelihood method. [file 1471-2229-10-128-S2.DOC]
