# Supplementary material for: Characterization of SQUAMOSA-like genes in Gerbera hybrida, including one involved in reproductive transition
Source: BMC Plant Biol. 2010 Jun 25;10:128. doi: 10.1186/1471-2229-10-128 (PMC3017819; doi:10.1186/1471-2229-10-128)
Supplement: Additional file 3 — Expression of GSQUAs during ray flower petal development. RNA gel blots showing the expression of GSQUA2, GSQUA3, GSQUA4, GSQUA5, and GSQUA6 at different stages of Gerbera ray flower development. [file 1471-2229-10-128-S3.PPT]

## Slide 1
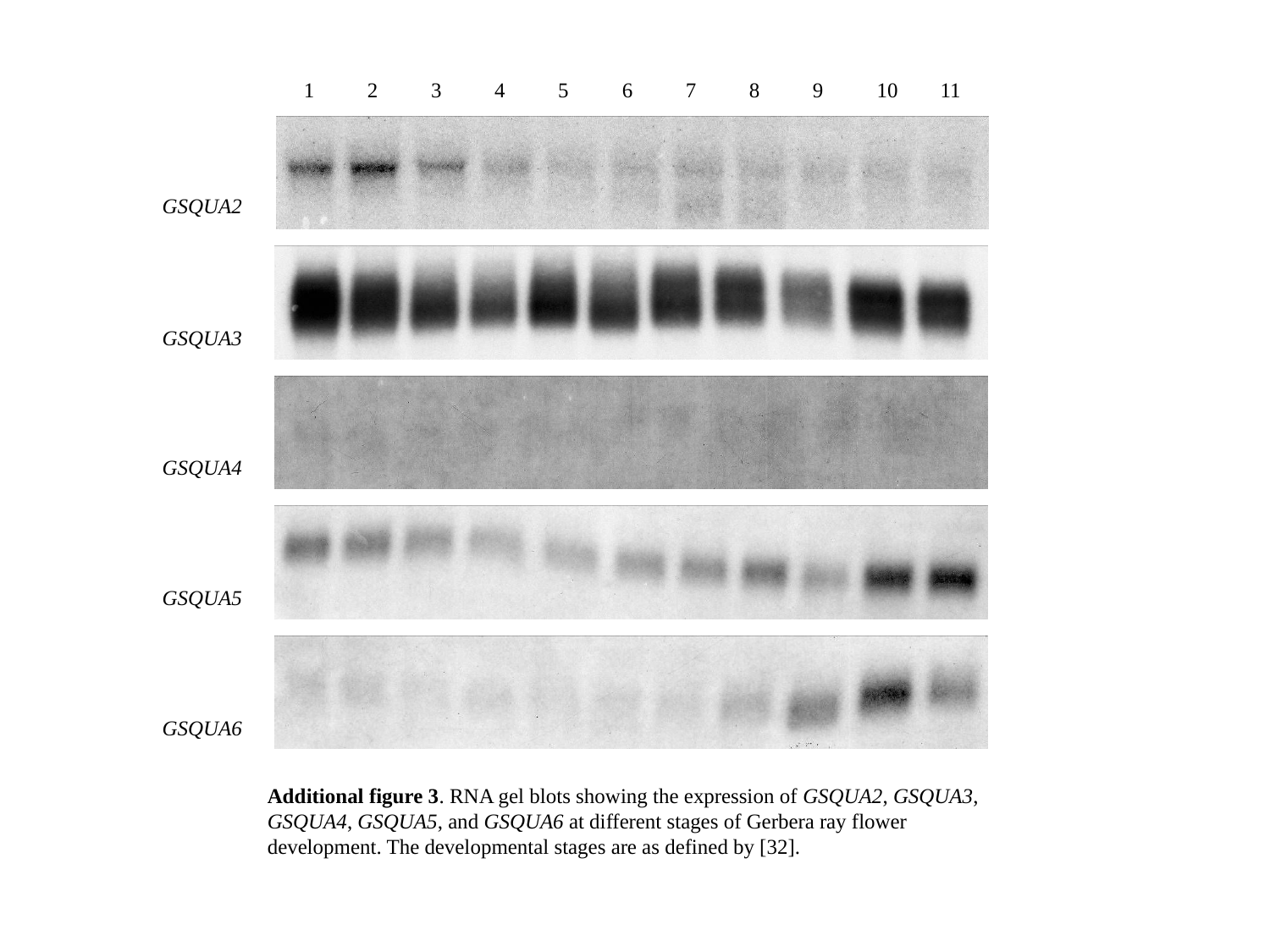

1
2
3
4
5
6
7
8
9
10
11
GSQUA2
GSQUA3
GSQUA4
GSQUA5
GSQUA6
Additional figure 3. RNA gel blots showing the expression of GSQUA2, GSQUA3, GSQUA4, GSQUA5, and GSQUA6 at different stages of Gerbera ray flower development. The developmental stages are as defined by [32].
