# Supplementary material for: Characterization of SQUAMOSA-like genes in Gerbera hybrida, including one involved in reproductive transition
Source: BMC Plant Biol. 2010 Jun 25;10:128. doi: 10.1186/1471-2229-10-128 (PMC3017819; doi:10.1186/1471-2229-10-128)
Supplement: Additional file 4 — Transgenic lines overexpressing GSQUA2. RNA gel blots showing GSQUA2 overexpression in transgenic lines. [file 1471-2229-10-128-S4.PPT]

## Slide 1
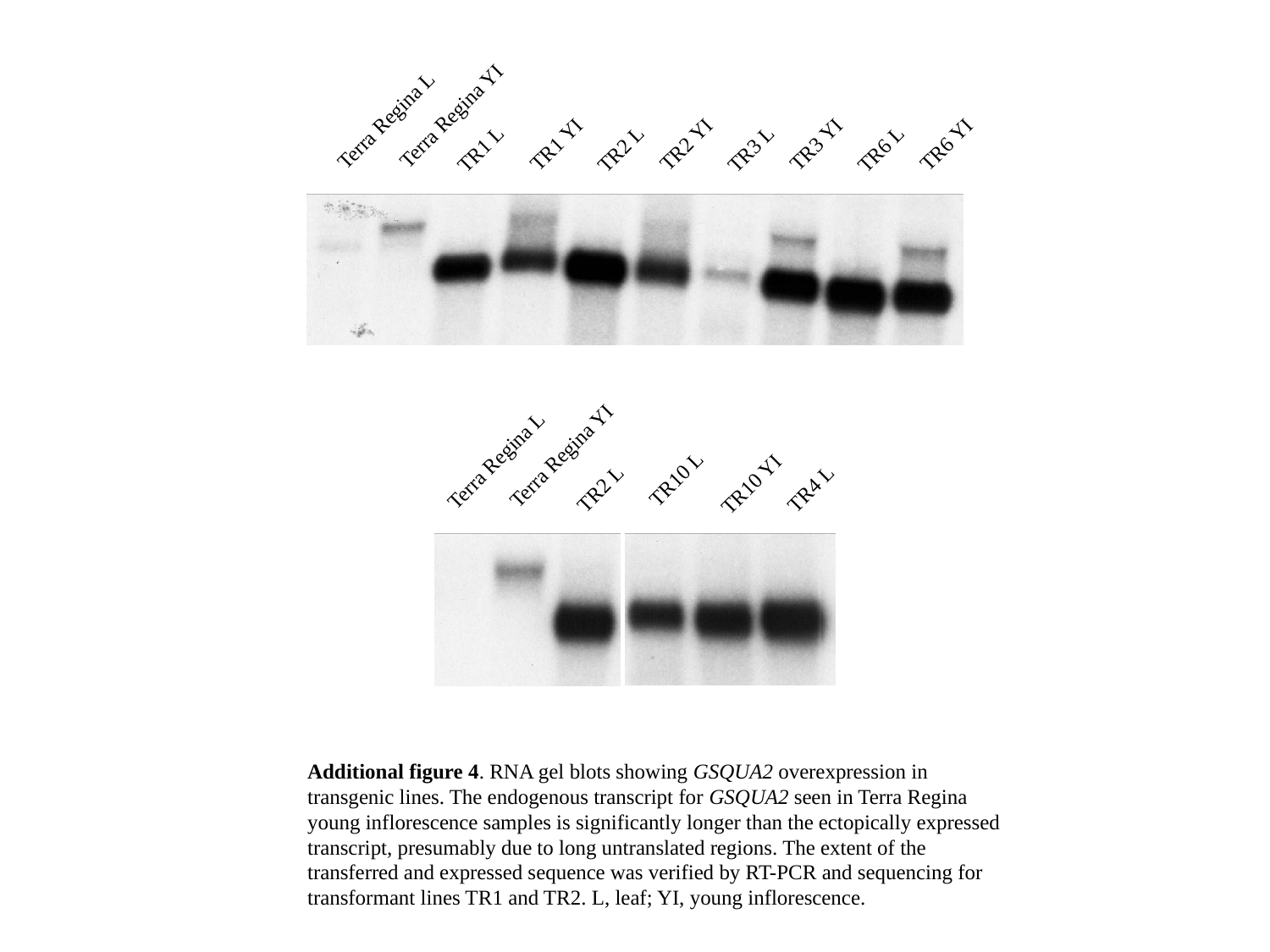

Terra Regina YI
Terra Regina L
TR1 YI
TR2 YI
TR3 YI
TR6 YI
TR6 L
TR1 L
TR2 L
TR3 L
Terra Regina YI
Terra Regina L
TR10 L
TR10 YI
TR4 L
TR2 L
Additional figure 4. RNA gel blots showing GSQUA2 overexpression in transgenic lines. The endogenous transcript for GSQUA2 seen in Terra Regina young inflorescence samples is significantly longer than the ectopically expressed transcript, presumably due to long untranslated regions. The extent of the transferred and expressed sequence was verified by RT-PCR and sequencing for transformant lines TR1 and TR2. L, leaf; YI, young inflorescence.
